# A framework of generating explanation for conceptual understanding based on “semantics of constraints”

**DOI:** 10.1007/s41039-015-0002-4

**Published:** 2015-06-23

**Authors:** Tomoya Horiguchi, Takahito Tomoto, Tsukasa Hirashima

**Affiliations:** 1 Graduate School of Maritime Sciences, Kobe University, 5-1-1, Fukaeminami, Higashinada, Kobe, Hyogo 658-0022 Japan; 2 Faculty of Engineering Division II, Tokyo University of Science, 1-3, Kagurazaka, Shinjyuku, Tokyo, 162-8601 Japan; 3 Department of Information Engineering, Hiroshima University, 1-4-1, Kagamiyama, Higashihiroshima, Hiroshima 739-8527 Japan

**Keywords:** Science education, Problem practice, Conceptual understanding, Explanation generation, Semantics of constraints

## Abstract

In science education, conventional problem practice hardly helps students reach “conceptual understanding” with which they can solve various problems by making appropriate models of target systems. Students often superficially read the solution of a problem and apply it wrongly to others without understanding the model. It is difficult to teach how to make appropriate models because model-making expertise includes a lot of implicit knowledge. In this paper, we propose a general framework for systematically describing such knowledge, which makes it possible not only to explain various models and the difference between them but also to design/sequence a set of problems appropriate for promoting conceptual understanding. Our framework was proved useful through a preliminary experiment in which the explanations generated based on our framework promoted subjects’ (15 graduates and undergraduates) conceptual understanding in mechanics. The framework can be the basis for designing intelligent tutoring systems which explicitly help students reach conceptual understanding.

## Introduction

In science education, it is one of the most important goals for students to acquire the ability to make an appropriate model of the target system and its behavior for a given task. The method most frequently used for achieving this goal is the “problem practice,” in which students are required to make an appropriate model for answering the query in each of a set of problems. They need to identify the structure/state of the target system and the applicable principles/laws for modeling the given situation. We call such ability “conceptual understanding” of a domain. (Note that models are not limited to mathematical ones because not a few problems are solved with non-mathematical models such as qualitative models).

However, it is difficult to reach such an understanding with conventional problem practice. In conventional problem practice, after learning some worked examples, students solve a set of problems one by one. Explanations about the solution mainly focus on the calculation of the required amount from the given ones and rarely focus on the reason why the solution is possible, whether and why the solution is/is not applicable to other problems. Problems are rather arbitrarily sequenced, and the relations between (the solutions of) problems are rarely explained. In such a practice, there are many students who wrongly apply the solution they previously learned (in one problem) to another in which the solution is inapplicable. There are also many students who cannot apply the solution they previously learned to another in which the solution is applicable. Even experience in many problems does not easily improve such difficulties (Bransford et al. 2000; VanLehn 1998; VanLehn and van de Sande 2009). Novice students often generate a naive representation of a problem focusing on its superficial features (called “surface structure” (Chi et al. 1981; Hirashima et al. 1994)). They cannot generate the representation of a problem based on its structural features (called “physical structure”) (Chi et al. 1981; Larkin 1983, 1985). Therefore, instead of applying principles/laws to make the model appropriate for each problem, they often try an inappropriate solution, such as to apply the solution of a problem to another (just as it is) based on their superficial similarity (VanLehn 1998) or to use a general strategy for operating mathematical equations without considering their physical meanings (Larkin 1981). This is mainly because they sometimes succeeded in solving problems with an inappropriate solution by accident in their past practice.

In order to reach conceptual understanding, students therefore need to learn (1) to infer the structural features of problems from the superficial features and (2) to apply appropriate principles/laws to structural features to make models necessary for solving problems. For assisting them, in problem practice, it is necessary to explain not only how each problem is solved but also why the solution is possible and what physical meaning it has. That is, it must be explicit why the principles/laws are applicable to the given situation (i.e., surface structure) and what physical meaning (physical structure) they imply. Additionally, it is important to explain not only the solution of a problem but also the relation (difference) between problems, that is, how the solution (applicable principles/laws) changes when the situation (problem) is changed. Furthermore, it would promote such learning to provide students with an appropriately designed and sequenced set of problems (Scheiter and Gerjets 2002, 2003; VanLehn and van de Sande 2009).

In conventional problem practice, such instruction has been rarely focused on, at most given by a few (experienced) teachers individually and implicitly. Especially, there have been few intelligent tutoring systems (ITS) which can explain the relation between arbitrary two problems and adaptively sequence problems considering the learning effect of order. We think this is because most of the knowledge necessary for such instruction is implicit and difficult to systematize; therefore, there has been no general framework for indexing various types of problems.

In this paper, we propose a general framework for indexing problems. The framework helps authors index problems. Based on such indices, the explanation and sequence of problems mentioned above can be automatically generated. In our framework, making a model in physics is regarded as a process in which various constraints (applied principles/laws and modeling assumptions) are imposed on the target system and its behavior. A model is regarded as the set of constraints. We first formulate the model-making process in physics, then analyze the constraints which compose a model to systematize them based on their physical meanings and roles (functions). After that, we describe the applicable conditions of principles/laws in physics as a set of constraints. The constraints classified/defined in this manner are easily assigned to the situation of a problem. There are also some groups of constraints which are “exclusive” from each other (i.e., cannot be valid simultaneously). Therefore, based on such classification and exclusiveness of constraints, it becomes possible to explain what physical meaning (structural features) superficial features of a problem have, what principles/laws are applicable to them, and how applicable principles/laws change when the situation is changed. By indexing problems with this framework (we call it “semantics of constraints: SOC”), it becomes possible to automatically extract the “differences between problems” necessary for the comparison and sequencing of problems.

The technical contributions of this paper are that a “system of concepts” is developed with which the expertise in solving physics problems can be explicitly described and that an explanation generator is developed which automatically generates explanations based on such descriptions. It is well known that “knowledge engineering” is necessary to represent experts’ knowledge on computers. Ikeda et al. pointed out such engineering (they call it “ontology engineering”) is also important in designing educational systems (Ikeda et al. 1999). We identified various types of modeling assumptions and other constraints (also the relations among them) which constitute models of physical systems and behaviors. Such a system of concepts (or ontology) works as a “conceptual tool” (Ikeda et al. 1999) which assists/guides authors in describing teaching materials (in our case, experts’ model-making process). Without our framework, it would be difficult for authors to describe such expertise explicitly.

The SOC framework originally arose from our research on “error-based simulation (EBS)” (Hirashima et al. 1998; Horiguchi and Hirashima 2001, 2006; Horiguchi et al. 2014). EBS is an educational simulation which is generated with students’ erroneous formulation (i.e., model) of physical systems (Hirashima et al. 1998). In EBS, students observe the behavior of systems which is unnatural/different from the correct one, so they are much motivated to correct their model. That is, EBS works as a counterexample to students’ erroneous ideas (Horiguchi and Hirashima 2001). Here, how the behavior is different from the correct one is important because students do not become aware of their errors if the difference is small. We therefore developed a set of heuristics with which it is judged whether an EBS is useful or not for error awareness (Hirashima et al. 1998; Horiguchi and Hirashima 2001). We also developed a simulator which can identify the constraint violation in the students’ erroneous model and relax some constraints (if necessary) to make the model calculable (Horiguchi and Hirashima 2006). The simulator (called “robust simulator”) uses a set of heuristics for judging what constraint(s) should be relaxed to generate (the most) educationally useful EBS. EBS has been proved useful for correcting students’ misconception through practical use in the classroom (Horiguchi et al. 2014). While the above heuristics were domain-dependent and had the limitation on their applicable area, the SOC framework is the generalization/elaboration of them. Recently, we used the framework to generate feedback to erroneous models the students made in a model-building learning environment (Horiguchi et al. 2012). By using the framework, what constraints are violated in students’ erroneous models is explained and how to correct their models is suggested.

We first discuss the required knowledge and assistance necessary for conceptual understanding based on current research, then introduce the SOC framework. After that, we show the method for generating SOC-based explanations. The results of the preliminary experiment are described which proved the usefulness of our framework. Finally, we conclude this paper and mention our future work.

## Conceptual understanding and assistance

Research on problem solving has revealed the knowledge structure domain experts (Chi et al. 1981; Larkin 1981, 1983, 1985; VanLehn 1998; VanLehn and van de Sande 2009) in science have. Experts can infer the structural features of problems with scientific concepts from the superficial features and generate the representation to which formal operations are applicable. They can also generate an appropriate plan for solving the problem by operating the representation with the knowledge about qualitatively interpreted principles/laws. It is supposed that experts have acquired such knowledge by inducing the essential features through comparison of many problems and by transforming them into (some kinds of) “schemata” or “production rules” (VanLehn and van de Sande 2009). It is, however, difficult for students to reach such an understanding through conventional problem practice. Even instructional innovations based on recent learning science research have limitedly improved students’ understanding (Bransford et al. 2000).

In order to promote such knowledge acquisition, it is effective to appropriately design a set of problems which includes positive/negative examples of various problem categories and “near misses” (near misses are minimally contrasting pairs of examples: one is positive and the other is a negative example of a category, and they differ in only one critical feature), and to provide them in an appropriate order to students (VanLehn and van de Sande 2009) (in fact, it is reported that problem order greatly influences learning (Scheiter and Gerjets 2002, 2003)). In order to do that, it is necessary to explicitly describe the superficial/structural features of problems and their relations, and qualitative interpretations of principles/laws and their means of application. However, since most of such knowledge is implicit, there has been no general framework to systematically describe such knowledge. We think this is the reason of the following fact: Though knowledge structure necessary for expertise was revealed and effective instructional methods were proposed, they have not been widely practiced. The framework we propose makes it possible to systematically describe such knowledge, based on which the design of a set/sequence of problems and explanation generation for promoting conceptual understanding become possible.

## Semantics of constraints

Given a physics problem (which consists of a physical system and query), one makes a model necessary and sufficient for answering the query by embodying an appropriate part of the domain theory. (A “necessary and sufficient” model means that it includes the information necessary for solving the problem and that it does not include the unnecessary (too detailed) information.) Domain theory (Choueiry et al. 2005; Conati and VanLehn 1996; VanLehn and van de Sande 2009) consists of a set of propositions, each of which describes a principle/law, its applicable condition, and resulting constraint(s) on the attribute(s) of the system. Constraints by embodied principles/laws are called the “physical phenomenon constraints (PPCs).”

In making a model, various modeling assumptions are set for selecting appropriate principles/laws. Modeling assumptions define the structure/behavioral range of a system and physical phenomena to be considered. Since embodied physical phenomenon constraints are valid under some modeling assumptions, applicable conditions of principles/laws can be described with a set of modeling assumptions. That is, a PPC always has its corresponding modeling assumptions. Constraints by modeling assumptions are called the “modeling assumption constraints (MACs).”

Boundary condition of a system is given by the “boundary condition constraints (BCCs).” They define the influence from the outside of the system. Making the influence which cannot/need not be calculated with a model given means defining the boundary of the model (i.e., what physical processes are considered/ignored). That is, a BCC always has its corresponding modeling assumptions.

In our framework, a model is the union of physical phenomenon constraints, boundary condition constraints, and modeling assumption constraints. Usually, only the first two constraints are written as a model while the last constraints are remained implicit. However, MACs give the validity to PPCs and BCCs. When modeling assumptions are changed, physical phenomena and boundary conditions also qualitatively change. In order to make a model correctly, it is therefore necessary to understand the physical meaning of the constraints based on modeling assumptions (i.e., why an assumption is set and what role it plays). In most cases, such knowledge is acquired by a few students. In this research, we develop a framework for describing such knowledge explicitly, based on which the function for promoting conceptual understanding is designed. In the following three subsections, we elaborate on each class of constraints to systematize their physical meanings and relations.

### Modeling assumption constraints (MACs)

Modeling assumption constraints define the physical processes considered/ignored in a model. They are classified in two ways from different viewpoints: structural and functional.

The structural viewpoint focuses on defining the structure and its state of a model. The “physical structure constraint” specifies what kind of objects, relations, and their attributes in a system are considered. It corresponds to selecting a viewpoint, granularity, or coordinate system of a system. It has two subclasses: “physical object constraint” and “physical attribute constraint.” The former specifies the objects to be considered. An example is the specification about whether two blocks in contact are considered as they are (two blocks) or as one block. The latter specifies the attributes/relations of objects to be considered. An example is the specification about whether a block’s mechanical attributes (e.g., mass, applied forces) or its electrical attributes (e.g., current, resistance) are considered. Another example is the specification about whether the friction between two blocks is considered or not. On the other hand, the “operating range constraint” specifies the range within which a model is valid since physical phenomena occur assuming a system is in a specific state. It has two subclasses: “physical range constraint” and “conceptual range constraint.” The former specifies the range which is defined with a (set of) physical amount(s). For example, a model of a resistance assuming its value is constant needs the specification that its current and voltage are within the proportional range. The latter specifies the range which is difficult to be defined with a (simple combination of) physical amount(s). For example, the model “an object in the water of a pond” requires the range of “in the water,” but it is difficult (or unnecessary) to be precisely defined if the pond has a complicated shape. The subclasses, definitions, and examples of modeling assumption constraints from the structural viewpoint are summarized in Table 1.

**Table 1:** Classes of modeling assumption constraints (from structural viewpoint)

	Subclasses	Subsubclasses	Definition	Examples
MAC	Physical structure constraint (PSC) Decision about the perspective and granularity in modeling	Physical object constraint (PO)	Specifies what kind of objects in a physical system are considered	- Consider two blocks in contact as they are or as one
				- Consider two parallel-connected springs/resistors as they are or a compound one
		Physical attribute constraint (PA)	Specifies what kind of relations/attributes of objects in a physical systems are considered	- Consider a block’s net-force or its electric resistance
				- Consider the friction between two objects in contact or not
	Operating range constraint (ORC) Decision about the behavioral range of the model	Physical range constraint (PR)	Specifies the range (state space) within which the model is valid by using physical attributes	- A model of two blocks’ motion where one pulls another thru a string assuming the string is taut
				- A model of a constant resistance assumes its current and voltage are within the proportional range
		Conceptual range constraint (CR)	Specifies the range (state space) within which the model is valid by using conceptual attributes	- A model of a block (*b*) descending a slope (*p*) by gravity from the gravitational field (*g*) assuming their positional relations are in (*b*, *g*), on (*b*, *p*)

The functional viewpoint focuses on defining the boundary of a model to specify what kind of physical processes is considered/ignored. The “process consideration constraint” makes such selection about physical processes of the same granularity (where the “out-sourcing/black-boxing constraint” ignores a physical process by putting it out of the system or into a black box regarding its effect as a boundary condition, and the “process selection constraint” simply ignores a physical process and its effect). For example, assuming constant voltage supplied from outside is an out-sourcing constraint. Considering two parallel-connected resistors as a compound resistor is a black-boxing constraint. Considering/ignoring the friction between two objects is a process selection constraint. The “physical world constraint” maintains the fundamental laws of the physical world, such as “rigid objects never overlap.” More microscopic physics is necessary to explain why this constraint is valid, that is, it specifies the physical processes of smaller granularity are ignored. The “process simplifying constraint” substitutes the simplified process for an original complicated process in order to make the (mainly mathematical) calculation with a model easier. An example is to consider the behavior of a pendulum with small amplitude as simple harmonic oscillation (not as circular motion). The subclasses, definitions, and examples of modeling assumption constraints from the functional viewpoint are summarized in Table 2.

**Table 2:** Classes of modeling assumption constraints (from functional viewpoint)

	Subclasses	Subsubclasses	Definition	Examples
MAC	Process consideration constraint (PCC) Decision about what kind of physical processes are considered in modeling	Process selection constraint (PS)	Specifies what kind of processes in a physical system are considered/ignored	- Consider (rel-friction(*b*_l_, *p*_l_)) PA
				- Ignore the change of form/mass of objects which collided PO
				- Consider the heat exchange between two objects (*T*_H_ > *T*_L_) PR
		Out-sourcing constraint (OS)	Ignores a physical process by putting it out of the system and regarding its effect as a boundary condition	- An outer tank which supplies water infinitely PR
				- An outer power supply which always supplies 5 V PR
				- Initial velocity *v*(0) = *v*_0_ PR
		Black-boxing constraint (BB)	Ignores a physical process by putting it in a black box and regarding its effect as a boundary condition	- Consider two parallel-connected springs/resistors as they are or a compound one PO
				- Consider two blocks in contact moving with internal force as one PO/CR
	Physical world constraint (PWC) Necessity for maintaining the fundamental law of the physical world		Maintains the fundamental law of the physical world	- Rigid objects never overlap CR
				- Mass >0, *μ* > 0, 0 ≤ *e* ≤ 1 PR
				- *g* = 9.8 [m/s/s], gas constant = 8.3 [J/K mol] PR
	Process simplifying constraint (PFC) Convenience for mathematical calculation with the model		Substitutes the simplified process for a complicated (original) process	- Consider the range within which a resistance’s voltage and current are proportional PR
				- Consider the behavior of a pendulum with small amplitude as simple harmonic oscillation PR

Constraint classes from the structural viewpoint are useful for enumerating modeling assumptions because they rather suggest the components and their relations of a system. For example, when a variable in an equation stands for a physical quantity, it is easy to infer an object and its attribute corresponding to the quantity is considered (which are physical structure constraints). Constraint classes from the functional viewpoint are useful for considering the meaning of modeling assumptions because they rather suggest the process structure (processes considered and their relations). For example, considering/ignoring a physical attribute (which is a physical structure constraint) suggests a physical process concerning the attribute is considered/ignored (which is a process selection constraint). That is, the classes from the structural viewpoint rather concern the surface structure of a problem, while the classes from the functional viewpoint rather concern its physical structure. Furthermore, as shown above, the classes from both viewpoints are related to each other based on their physical meanings. Therefore, with these classifications, it becomes possible to systematically describe the knowledge about the relation between superficial and structural features of problems.

Additionally, there are often sets of modeling assumption constraints which are mutually exclusive (cannot be assumed simultaneously). For example, in the same time interval, “transient state” and “steady state” (which are operating range constraints) cannot be assumed simultaneously. In the same (part of a) system, “consider friction” and “not consider friction” (which are process consideration constraints) cannot be assumed simultaneously. Such exclusiveness between modeling assumptions gives important clues to identify the differences between models/problems (see the Procedure section).

### Physical phenomenon constraints (PPCs)

A relatively simpler physical phenomenon constraint is the “physical device constraint” which arises within a component of a system. That is, it is a “local constraint.” Since it indicates the physical property of the component, each domain has its specific physical device constraints (for example, Ohm’s law constrains the values of current and voltage in an electric device). In contrast, there are “global constraints” which indicate the behavior of multiple components or the whole system. Global constraints are classified as follows.

In general, a physical system evolves through time, starting from an initial state. It is either (1) changing dynamically, (2) in a steady state, or (3) changes discontinuously. Therefore, we call the constraints in these states the “dynamic change constraint,” the “steady state constraint,” and the “discontinuous change constraint,” respectively. Additionally, when a quantity is conserved through time, the constraint which indicates its amount is the same at arbitrary two time points is called “conservation law constraint.” The subclasses, definitions, and examples of global physical phenomenon constraints are summarized in Table 3.

**Table 3:** Classes of physical phenomenon constraints

	Subclasses	Subsubclasses	Definition	Examples
PPC	Dynamic change constraint (DYC)	Differential change constraint (DC)	Specifies the behavior of a physical system while it is dynamically changing (with differential expression)	- *F* = Ma − *T*
				- *Ld* ^2^ *q*/*dt* + *Rdq*/*dt* + (1/*C*)*q* = *V*_*s*_
		Integral change constraint (IC)	Specifies the behavior of a physical system while it is dynamically changing (with integral expression)	- *x*(*t*) = *x*_0_ + *v*_0_ *t* + (1/2)*at* ^2^
				- *T* = 2*π*√ (*m*/*k*)
				- *i*(*t*) = *CV*_*s*_ *αβ*(*β* − *α*){*e* ^*βt*^ − *e* ^*αt*^}
	Steady state constraint (SSC)		Specifies the behavior of a physical system in a steady state	- ∑*F*_*i*_ = 0
				- *Q*_in_ = *Q*_out_
	Discontinuous change constraint (DCC)		Specifies the behavior of a physical system when it changes discontinuously	- (*v*_1_ ' − *v*_2_ ')/(*v*_1_ − *v*_2_) = *e*
				- *i* = 0 (*v*_*D*_ < 0), *i* ≥ 0(*v*_*D*_ = 0)
	Conservative law constraint (CLC)		Specifies the amount of a quantity which is conserved over the time	- (1/2)*mv* ^2^ + *mgh* = const.
				- *m*_1_ *v*_1_ + *m*_2_ *v*_2_ = const.
				- *A*_*m*_ (water_1_) = *A*_*m*_ (water_2_) = const.

The dynamic change constraint constrains the behavior of a system in a time interval during which it is changing dynamically. It has two subclasses: “differential change constraint” and “integral change constraint.” The former often indicates the relation between the *driving power* of dynamic change and the influences on it, and is represented with differential expression. For example, Newton’s second law (equation of motion) relates an object’s acceleration with the forces applied to it. The latter usually includes a time variable and describes the temporal/integral effect of the driving power, and is represented with integral expression. An example is the expression of linear accelerated motion. The steady state constraint constrains the behavior of a system in a time interval during which it is in a steady state. It indicates the balance/cancelation between influences on the driving power of dynamic change. An example is the equation of balance of forces about an object at rest. The discontinuous change constraint constrains the behavior of a system at a time point on which it changes discontinuously. It indicates the relation between the amounts of a quantity before and after the change. An example is the formula of coefficient of restitution. A quantity is called a “conserved quantity” when its amount is constant during the temporal evolution of a system. The conservation law constraint indicates the amounts of a conserved quantity at arbitrary two time points are the same. An equation of heat exchange between two objects and an equation of conservation of energy/momentum are the examples.

A global physical phenomenon constraint aggregates a set of local physical phenomenon constraints. For example, Newton’s second law (equation of motion), which is a dynamic change constraint in mechanics, includes a set of local constraints, each of which indicates a force applied to the target object (physical device constraints such as elastic force, friction). Such inclusion relation between PPCs gives important clues to identify the dominant principle(s)/law(s) in solving a problem.

Additionally, there are often sets of physical phenomenon constraints of which modeling assumptions (preconditions) are mutually exclusive. These PPCs are never simultaneously valid in the same state of the same system. For example, since “static friction” and “kinetic friction” have exclusive preconditions (operating range constraints) about a contact surface of two objects, they are never valid simultaneously at the same surface. The first three global PPCs (i.e., dynamic change, steady state, and discontinuous change constraints) are exclusive for the same reason. They often entirely change each other when preconditions are changed. For example, suppose a mechanical system is in a steady state by assuming “friction” which cancels other forces. When the assumption is changed to “frictionless,” the system can dynamically change. Such exclusiveness between PPCs gives important clues to identify the differences between models/problems (see the Procedure section).

### Boundary condition constraints (BCCs)

Since the outside of the structure and behavioral range of a system defined with modeling assumptions are not modeled, it is necessary to define the influence from the outside at the boundary in order to calculate the behavior of the system. BCCs give such influence, that is, the boundary values of a set of physical attributes (of objects, relations, and their compound amounts). Therefore, considering BCCs gives important clues to understand the role of corresponding modeling assumptions in defining the system boundary.

A boundary condition is usually given to an attribute at a time point of system behavior. If the value of the attribute is defined as temporally constant (not variable), the constraint by the boundary condition continues during a time interval. Such constraint is called “constant boundary constraint” (the attribute is called “constant attribute”). A boundary condition which does not temporally continue is called “variable boundary constraint” (the attribute is called “variable attribute”). The former suggests a physical process is ignored which influences the attribute to change its initial value, while the latter suggests such a physical process is considered (i.e., process selection constraints). Both constraints suggest the history is ignored through which an attribute got its initial value (i.e., out-sourcing constraint). For example, when the mass of an object is given as a constant, the physical processes such as “collision” and “corrosion” are ignored which would change its value. When the initial velocity of an object is given (as a variable), the process is out-sourced through which the object got the velocity. The subclasses, definitions, and examples of boundary condition constraints are summarized in Table 4.

**Table 4:** Classes of boundary condition constraints

	Subclasses	Definition	Examples
BBC	Constant boundary constraint (CBC)	Specifies the boundary condition (initial value) of temporally constant attribute	- Mass(*B*_1_) = *M*, spring-const(*S*_1_) = *K*
			- g-acc(GF) = 9.8 [m/s/s]
			- In (*B*, GF) (object *B* is always in gravity field GF)
			- Resistance(*R*_1_) = *R*
			- Compound-capacitance(*C*_1_, *C*_2_) = *C*
	Variable boundary constraint (VBC)	Specifies the boundary condition (initial value) of temporally variable attribute	- vel(*B*_1_,*t*_0_)
			- Compressed(*S*_1_,*t*_0_) (spring *S*_1_ is initially compressed)
			- on(*B*, *S*, *t*_0_) (object *B* is initially on slope *S*, but *B* might leave *S*)
			- Variable-resistance(*R*_1_, initial) = *R*_init_

Additionally, a BCC sometimes suggests the behavior of a system is assumed to be within a specified behavioral range (operating range constraint). For example, when considering the motion of an artificial satellite, its initial velocity is usually given as it maintains the circular orbit around the earth.

## Explanation generation

### Framework of model-making process description

In our framework, each principle/law is described as a “model fragment” (Falkenhainer and Forbus 1991) which consists of its applicable condition and its consequence(s). An applicable condition is described as a set of modeling assumption constraints, while a consequence is described as a physical phenomenon constraint. A model consists of the union of PPCs given by instantiated model fragments, MACs giving applicable conditions for them, and BCCs given in a problem. (Note that an instantiated “model fragment” is distinguished from “model fragment class” which describes a principle/law itself.) A model-making process (i.e., solution) is described as the procedure in which model fragments are applied (instantiated) in turn to the situations (represented with MACs and BCCs) to yield new consequences (represented with PPCs). (Note that a consequence of a model fragment can be the condition for others.)

**Fig. 1:**
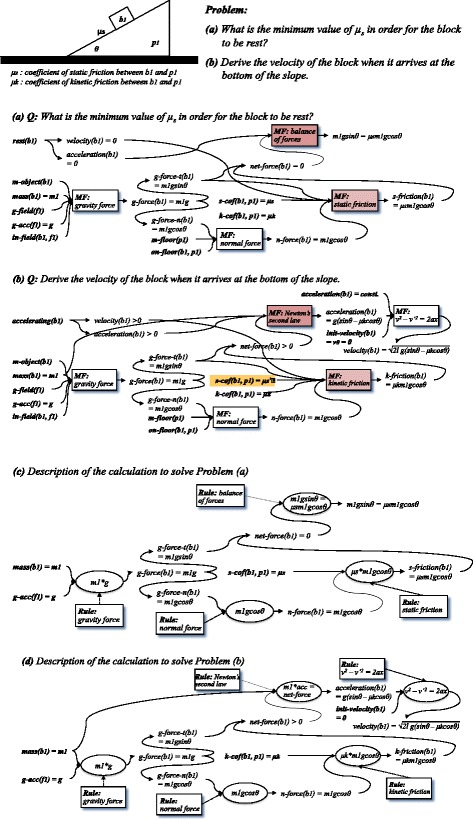
Examples of model-making process

Figure 1a,b shows examples, in which it is explicitly described why/how each principle/law is applied to the given situation. In contrast, the usual description of a solution focuses on the calculation of the required physical amount from the given ones, while the principles/laws and conditions which justify the calculation are attached in the ad hoc way. Figure 1c,d are typical descriptions of such solution we described according to the format of “solution graph” (Conati and VanLehn 1996) of ANDES (VanLehn et al. 2005). They are the solutions of problems (a) and (b), respectively. SOC enables implicit assumptions and physical meanings of calculation to be systematically described.

For example, in Fig. 1a, the constraint “the block b1 is on the inclined floor p1 (on-floor(b1, p1))” is explicitly described. It is an important condition under which the normal force (*n-force(b1)*) is exerted on the block by the floor. In contrast, in Fig. 1c, since this constraint is not described, it cannot be inferred that the solution becomes invalid when the inclination of the floor exceeds 90° (i.e., the block is no longer “on the floor”). For another example, the constraint on the initial velocity of the block (*velocity(b1) = 0*) is explicitly described in Fig. 1a while it is not in Fig. 1c. With the latter description, it cannot be inferred that the solution becomes invalid when the initial velocity of the block becomes greater than zero. These constraints are rarely explicitly described because they are usually implicit assumptions. Our framework can prompt the authors of the problem/solution to make such constraints explicit.

Additionally, a pair/set of model fragment classes which have similar conditions (situations) but have exclusive MAC(s)/PPC(s) as applicable condition(s)/consequence(s) is called “exclusive model fragment classes.” Grouping such model fragment classes helps the comparison between models.

For example, the model fragments “static friction” in Fig. 1a and “kinetic friction” in Fig. 1b are mutually exclusive because their applicable conditions (assumptions) “the net-force of the block in the tangent direction to the inclined floor is smaller than the maximum static friction” and “the net-force of the block in the tangent direction to the inclined floor is greater than the maximum static friction” are exclusive. Note that the constraint on the value of the coefficient of static friction (*s-cof(b1, p1) = μ*_*s*_ *’*) is described in Fig. 1b. It is not necessary for calculating the required amount, but is necessary for judging the block does descend the inclined floor. In the usual description of solution in Fig. 1d, since such a constraint is not explicitly described, it cannot be related to the other solution in Fig. 1c. Therefore, it cannot be inferred what happens when the value of the coefficient of static friction increases.

### Procedure

#### Explanation of the model-making process (solution)

The description of model-making process mentioned above makes it possible to explain why/how each principle/law is applied by explicitly referring to its modeling assumptions. In Fig. 1b, for example, the formula *v* ^2^ − *v*_0_^2^ *=* 2*ax′* (dynamic change constraint) is used which relates an object’s displacement, velocity, and acceleration in a time interval. Note that the constraint “acceleration is constant in the interval” (operating range constraint) is explicitly described which is an important precondition for this model fragment to be applied. Many students wrongly use this formula when an object’s acceleration temporally varies. The explanation explicitly referring to modeling assumptions would be helpful in avoiding such mistakes.

**Fig. 2:**
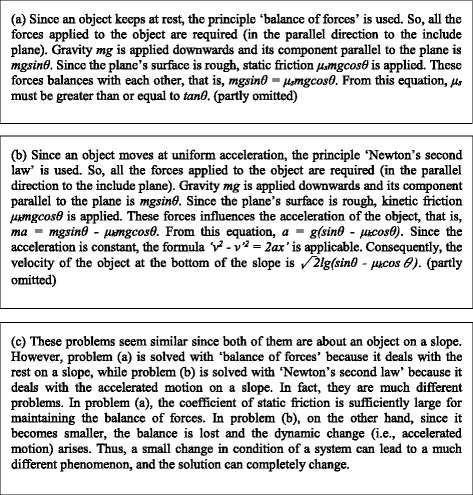
Examples of generated explanation

**Fig. 3:**
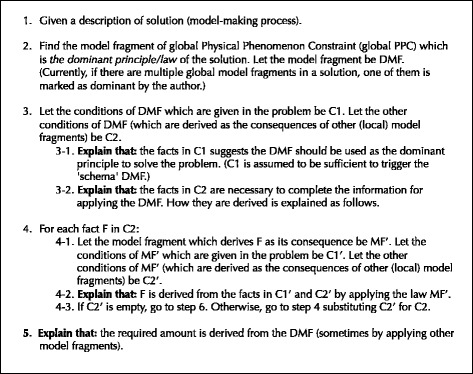
Procedure of generating explanation of a model-making process

Additionally, in solving problems, it is important to recognize not only each local principle/law and its consequence, but also the global principle/law which dominates the behavior of the whole system. The solution of domain experts is often “dominant principle/law-driven,” that is, they first recognize the dominant principle/law of a problem, then apply local principles/laws to “fill in the slots” of the global principle/law (Chi et al. 1981; Larkin 1983; VanLehn and van de Sande 2009). In our framework, a model fragment of global PPC is defined as the aggregation of the model fragments of local PPC which compose the global one (the applicable condition of a global model fragment is the union of its component model fragments). Global model fragments make it possible to explain the model-making process (solution) focusing on the dominant principle/law. For example, in Fig. 1a, the model fragment “balance of forces” gives a steady state constraint (global PPC), and its applicable condition includes some physical device constraints (local PPCs) given by other model fragments. Based on such inclusion relation, the sequence of explanation can be generated as follows: First, the explainer indicates that the given condition “a block is at rest” (which means its velocity does not temporally vary) suggests “balance of forces” should be used as the dominant principle, then the explainer refers to the laws “gravity” and “static friction” which influence the driving power of the block’s velocity. The generated explanation is shown in Fig. 2a. The explanation as to Fig. 1b is also shown in Fig. 2b. The procedure of the explanation generation is outlined in Fig. 3.

#### Explanation of the difference between models (problems)

The difference between models (problems) can be inferred by comparing their model fragments. There are two types of relations between problems: (1) the problems which have the same/similar surface structures (situations) but have different physical structures (instantiated model fragments belong to different classes) and (2) those which have different surface structures (situations) but have the same/similar physical structures (instantiated model fragments belong to the same classes). Both relations play an important role for promoting conceptual understanding (Scheiter and Gerjets 2002, 2003). As for the latter, the difference is easily inferred by identifying the corresponding pair of model fragments (each of which belongs to each model) both of which give the (global) PPCs of the same class. The difference can be explained by showing their preconditions (situations) are different.

As for the former, the difference is inferred by identifying the corresponding pair of model fragments (each of which belongs to each model) which belong to exclusive model fragment classes. Since their situations are similar but their modeling assumption constraint(s) and physical phenomenon constraint(s) are exclusively different, they indicate the difference of two models before/after the change of the situation. The type of the difference can be explained by referring to their modeling assumption classes. For example, when two corresponding model fragments have the same physical structure constraints and exclusively different operating range constraints, it is inferred that the difference of the two models is change of the operating range about the partial system they match. The model fragments “static friction” in Fig. 1a and “kinetic friction” in Fig. 1b are in such relation. It can be inferred that the local constraint between a block and slope is changed from “static friction” to “kinetic friction” by changing the operating range, by which the global constraint “balance of forces” (steady state constraint) is changed to “Newton’s second law” (dynamic change constraint) (the generated explanation is shown in Fig. 2c).

**Fig. 4:**
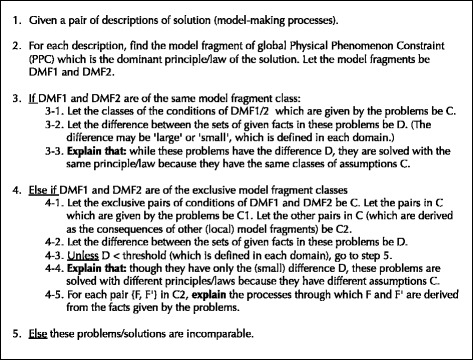
Procedure of generating explanation of a difference between models

Additionally, when comparing models (problems), it is important to recognize not only the change of each local principle/law and its consequence, but also the change of the global principle/law which dominates the behavior of the whole system. Global model fragments, which aggregate the model fragments of local PPCs, make it possible to explain the behavioral change of the whole system focusing on the dominant principle/law. The procedure of the explanation generation is outlined in Fig. 4.

## Preliminary experiment

### Design

We conducted an experiment to evaluate the usefulness of our framework. A SOC-based explanation generator was implemented. The purpose was to examine whether the SOC-based explanation promotes students’ conceptual understanding, that is, whether their representation of problems was improved and they became able to solve various types of problems by using correct models.

#### Subjects

Fifteen graduates and undergraduates whose majors are engineering participated.

#### Instruments

(1) Two sets of problems in elementary mechanics: They were called “problem set 1 (PS-1)” and “problem set 2 (PS-2).” Each set included 15 problems of various surface/physical structures. Problems might have similar situations but different solutions, or have different situations but similar solutions. The sets had no common problem. (2) Usual explanation about the solutions of 11 problems in PS-1: The calculation of the required physical amount from the given ones was mainly explained. (3) SOC-based explanation about the solutions of the same problems as usual explanation: In addition to the solution of each problem, the differences between problems were explained about eight pairs of problems which had similar surface/physical structures. (4) Explanation generator used for generating SOC-based explanation: Model-making processes described by the experimenter (first author) were input and their explanations were output, which were rewritten into readable natural language by the experimenter (without changing the point).

#### Procedure

First, subjects were given PS-1 and asked to group the problems into some categories based on some kind of “similarity” they suppose (any number/size of categories were allowed), then asked to label each category they made (called “categorization task 1”). After that, they were asked to solve eight problems in PS-1 (called “pre-test”). After a week, the subjects were divided into two groups: one was the “control group” (seven subjects) and another was the “experimental group” (eight subjects). The average scores of both groups in the pre-test were made equivalent. The subjects in control group were given the usual explanation and asked to learn it. The subjects in the experimental group were given the SOC-based explanation and asked to learn it. After that, by using PS-2, “categorization task 2” was conducted in the same way as above. Finally, subjects were asked to solve eight problems in PS-2 (called “post-test”).

#### Measure

The quality of the representation of problems was measured with the categories, their “frequencies” (number of problems accounted for), and the time required in each categorization task. The ability to solve various types of problems was measured with the scores in each test. The effect of learning with usual/SOC-based explanation on the quality of representation and the ability of problem solving was measured with the comparison of the results of two categorization tasks and pre-/post-tests. The superiority of the SOC-based explanation to the usual explanation was measured with the differences of improvement of categorization and problem solving between experimental and control groups.

## Results

The categories made by subjects and their frequencies in categorization task 1 are shown in Table 5. Most of the subjects categorized the problems based on the similarity of their superficial features, such as the components of the system (e.g., inclined plane, springs) and the figures of motion (e.g., circular motion, free fall). Additionally, all subjects finished the task within 10 min. The results of categorization task 2 are shown in Table 6 (for the control group) and Table 7 (for the experimental group). Many subjects of the control group still categorized the problems based on the similarity of their superficial features, while many subjects of the experimental group came to categorize the problems based on the similarity of their structural features, that is, the dominant principles/laws of problems (e.g., Newton’s second law, balance of forces, conservation of energy). Additionally, all subjects of the control group finished the task within 10 min again, while the subjects of the experimental group required from 25 to 35 min. These results suggest that the learning with the SOC-based explanation promoted representing problems based on their structural features rather than their superficial features (the increase of the time required suggests the subjects of the experimental group inferred the physical structure from surface structure).

**Table 5:** Categories in task-1

	Number of subjects using category labels (*N*_1_ = 15)	Average size of category (*N*_2_ = 15)	Number of problems accounted for (*N* = *N*_1_ × *N*_2_ = 225)	Number of problems wrongly accounted for (N* = 225)	Number of problems correctly accounted for (*N* ^C^ = *N* − N*)
*Springs*	12	3.1	37	2	35
*Free fall, etc.*	9	4.1	37	2	35
*Collision*	12	2.0	24	0	24
*Circular motion*	12	1.9	23	1	22
*Acceleration*	3	5.7	17	1	16
*Strings*	7	2.0	14	0	14
*Inclined planes*	5	2.2	11	0	11
Balance	5	2.4	12	4	8
Object only	1	5.0	5	0	5
Friction	3	1.7	5	0	5
Second law	2	2.5	5	2	3
Pulleys	1	2.0	2	0	2
Balance of energies	1	4.0	4	2	2
Motion of weight	1	2.0	2	0	2
Energy of falling object	1	3.0	3	2	1
Linear uniform motion	1	1.0	1	0	1
Simple harmonic motion	1	1.0	1	0	1
Terminal velocity	1	1.0	1	0	1
Internal force	1	5.0	5	5	0
Others	1	15	15	2	13

**Table 6:** Categories in task-2 (usual)

	Number of subjects using category labels (*N*_1_ = 7)	Average size of category (*N*_2_ = 15)	Number of problems accounted for (*N* = *N*_1_ × *N*_2_ = 105)	Number of problems wrongly accounted for (N* = 105)	Number of problems correctly accounted for (*N* ^C^ = *N* − N*)
*Springs*	4	4.5	18	0	18
*Inclined planes*	4	3.3	13	0	13
*Balance of forces*	3	3.7	11	0	11
Conservation of energy	3	6.0	18	9	9
Second law	3	3.7	11	2	9
Pulley and string	2	3.5	7	0	7
Circular motion	4	1.5	6	0	6
Pendulum	3	1.7	5	0	5
Simple harmonic motion	2	2.0	4	1	3
Collision	2	1.0	2	0	2
Inertial force	1	1.0	1	0	1
Friction	1	1.0	1	0	1
Others	2	4.0	8	1	7

**Table 7:** Categories in task-2 (SOC)

	Number of subjects using category labels (*N*_1_ = 8)	Average size of category (*N*_2_ = 15)	Number of problems accounted for (*N* = *N*_1_ × *N*_2_ = 120)	Number of problems wrongly accounted for (N* = 120)	Number of problems correctly accounted for (*N* ^C^ = *N* − N*)
*Balance of forces*	7	4.4	31	5	26
*Second law*	7	3.6	25	1	24
*Conservation of energy*	8	4.1	33	12	21
Linear accelerated motion	3	3.3	10	2	8
Conservation of momentum	3	1.3	4	1	3
Acceleration	1	3	3	0	3
Springs	1	3	3	0	3
Pulleys	1	3	3	0	3
Simple harmonic motion and period	2	1	2	0	2
String and tension	1	2	2	0	1
Time	1	2	2	0	2
Friction	1	1	1	0	1
Pendulum	1	1	1	0	1
Collision	2	1	1	0	1

**Fig. 5:**
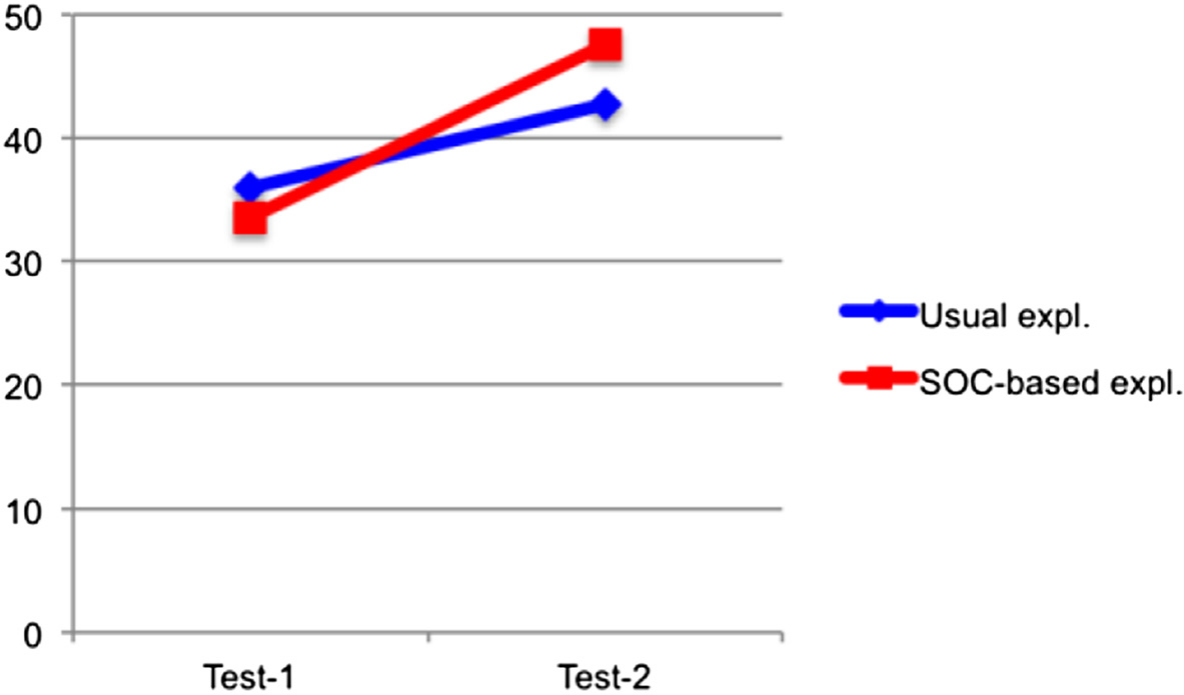
Average scores of tests

The average scores in pre- and post-tests are shown in Fig. 5 (in both tests, full marks were 52). In the pre-test, there was no significant difference of average scores between groups (control group 36.0 and experimental group 33.6, *t* test *p* > .10). In the post-test, though there was also no significant difference of average scores between groups (control group 42.7 and experimental group 47.6, *t* test *p* > .10), the increase of the average score of the experimental group was larger than that of the control group. This result suggests that the learning with the SOC-based explanation promoted the ability to solve various types of problems, that is, to make appropriate models regardless of their superficial features.

These results suggest that the SOC-based explanation about the solution of problems and their differences can assist students in reaching conceptual understanding.

## Conclusion

Aiming at promoting conceptual understanding through problem practice, we proposed the SOC framework, based on which the knowledge necessary for designing a set of problems, sequencing them, and generating explanations can be described.

Modeling assumptions play an important role in modeling because they give the physical meanings of the other constraints in a model (i.e., physical phenomenon and boundary condition constraints). Clarifying their relations makes it possible to generate schema-based explanation about a solution and the difference between solutions. As we discussed in the Explanation Generation section, the explanation generated by current ITS (Conati and VanLehn 1996; VanLehn et al. 2005; VanLehn and van de Sande 2009) does not focus much on modeling assumptions, or does so at most in an indirect/ad hoc way. (The research by Hirashima et al. (1994) was the pioneering work which tried to make such implicit knowledge explicit in ITS, but its domain was limited to mechanics.) This is because there was no framework for systematically describing modeling assumptions and the other constraints focusing on their roles in models/solutions. Giving such a framework is our contribution.

The importance of modeling assumptions in modeling was first indicated in the research on “compositional modeling” (Choueiry et al. 2005; Falkenhainer and Forbus 1991; Weld and deKleer 1989) in artificial intelligence. The purpose of compositional modeling is to automatically generate the model of a physical system by combining a set of model components (called model fragments) based on the modeling assumptions. While some important assumptions are identified in the research, we elaborated and systematized them into a framework especially aiming at education. Instead of automatic model generation, we enabled the description of problems/solutions with which explanation about experts’ problem solving process is automatically generated. We also enabled the design of a set of problems with which an appropriate sequence of problems can be automatically generated.

Automatic model generation by compositional modeling is not sufficient for automatic explanation generation in education. That is, in automatic model composition, an inference chain is generated which starts with the preconditions (given facts) and ends with the derived consequences (required amounts). However, sequencing the steps in the chain simply in one direction (either forward or backward) results in the explanation which is different from human experts’ explanation. As discussed in the Explanation Generation section, experts first identify the dominant principle/law in solving a problem, then apply other principles/laws to derive its conditions. That is, the explanation goes back and forth. Our explanation generator enables such an explanation by using the SOC-based description of solution. In fact, the description of the solution in our framework is “redundant” as an inference chain of automatic model composition. For example, in Fig. 1 (a), though the fact “acceleration(b1) = 0” is linked to the model fragment “balance of forces,” this fact is not necessary for the automatic application of this model fragment (only the fact “net-force(b1) = 0” is necessary). However, this link plays an important role in explanation generation because it represents experts’ insight “balance of forces is the dominant principle of the solution.” Since such a link must be written manually, it is our important future work to assist the authors in describing such information based on the SOC framework.

Such “redundant” information is also important for designing a set of problems. That is, for promoting conceptual understanding, it is important to show a small/large difference in surface features between problems does/does not lead to the difference in their solutions. Such critical features are often represented as the information which is redundant from the viewpoint of automatic model composition. For example, in Fig. 1a,b, what principle/law is dominant is identified not with the feature “net-force(b1) =/> 0” but with the feature “acceleration(b1) =/> 0” (the former is necessary for applying the model fragment “balance of forces/Newton’s second law” while the latter is not). Additionally, the difference between the (local) model fragments “static friction” and “kinetic friction” arises not because of the difference of the values of coefficients of friction but because of the difference of the dominant principle/law. Such information (i.e., “redundant” links and the weight (importance) of facts/model fragments) is important for comparing/designing a set of problems from the viewpoint of education, while it is not addressed in compositional modeling.

We showed the explanations generated with our framework could promote conceptual understanding through a preliminary experiment. Of course it is preferable to implement an authoring system which assists/guides authors in describing model-making processes with our framework. It is our future work. The SOC-based explanation generator can provide a basic function for designing various instructional methods (e.g., a detailed explanation is gradually simplified (scaffolding-fading), a sequence of problems is given which promotes spontaneous induction). Design of such instructional methods and verification of their effectiveness are also our future work. Additionally, as mentioned in the Introduction section, the SOC framework is developed based on the experience of developing/using intelligent tutoring systems in practice. Therefore, it has the potential to work as a conceptual basis in designing various intelligent functions of educational systems. Exploring such potential is our important future work.
